# Liquid Metal Particles–Graphene Core–Shell Structure Enabled Hydrogel-Based Triboelectric Nanogenerators

**DOI:** 10.3390/gels12010086

**Published:** 2026-01-19

**Authors:** Sangkeun Oh, Yoonsu Lee, Jungin Yang, Yejin Lee, Seoyeon Won, Sang Sub Han, Jung Han Kim, Taehwan Lim

**Affiliations:** 1 Division of Chemical Engineering and Bioengineering, Kangwon National University, Chuncheon 24341, Republic of Korea; 5sangkuen@kangwon.ac.kr (S.O.); yoonmul@kangwon.ac.kr (Y.L.); didwjddls32@kangwon.ac.kr (J.Y.); dpwls0320@kangwon.ac.kr (Y.L.); 2 Department of Chemical Engineering, University of Utah, Salt Lake City, UT 84112, USA; seoyeon.won@utah.edu; 3 Strategic Research Center for Smart Battery, Korea Basic Science Institute, Daejeon 34133, Republic of Korea; 4 Department of Material Science and Engineering, Dong-A University, Busan 49315, Republic of Korea

**Keywords:** hydrogel, graphene, liquid metals, redox reaction, triboelectric, nanogenerator

## Abstract

The development of flexible and self-powered electronic systems requires triboelectric materials that combine high charge retention, mechanical compliance, and stable dielectric properties. Here, we report a redox reaction approach to prepare liquid metal particle-reduced graphene oxide (LMP@rGO) core–shell structures and introduce into a poly(acrylic acid) (PAA) hydrogel to create a high-performance triboelectric layer. The spontaneous interfacial reaction between gallium oxide of LMP and graphene oxide produces a conformal rGO shell while simultaneously removing the native insulating oxide layer onto the LMP surface, resulting in enhanced colloidal stability and a controllable semiconductive bandgap of 2.7 (0.01 wt%), 2.9 (0.005 wt%) and 3.2 eV (0.001 wt%). Increasing the GO content promotes more complete core–shell formation, leading to higher zeta potentials, stronger interfacial polarization, and higher electrical performance. After embedding in PAA, the LMP@rGO structures form hydrogen-bonding networks with the hydrogel nature, improving both dielectric constant and charge retention while notably preserving soft mechanical compliance. The resulting LMP@rGO/PAA hydrogels show enhanced triboelectric output, with the 2.0 wt/vol% composite generating sufficient power to illuminate more than half of 504 series-connected LEDs. All the results demonstrate the potential of LMP@rGO hydrogel composites as promising triboelectric layer materials for next-generation wearable and self-powered electronic systems.

## 1. Introduction

The rapid advancement of electronics for the artificial intelligence (AI) era, with the development of wearable systems, has highlighted the growing need for sustainable power sources that do not rely on conventional batteries or external energy supplies [1,2,3]. Self-powered systems for electronic devices can offer cost-effective benefits and reduce the environmental burden associated with repeated battery replacement, thereby enabling more practical and eco-friendly operation in various applications [4,5,6,7]. Consequently, energy harvesting that is capable of converting and collecting mechanical energy, which is otherwise wasted, in the surrounding environment has thus emerged as a promising alternative [8,9]. Triboelectric nanogenerators (TENGs) have especially attracted significant attention due to their broad material compatibility, simple device architecture, ease of integration into wearable systems, and relatively high output performance [10,11,12,13]. These benefits have led to extensive research trends toward the development and optimization of TENGs.

TENGs operate based on the coupling effect of contact electrification and electrostatic induction, where the density of generated surface charges and the ability to retain these charges are key factors determining the device’s output performance. To enhance the output performance of TENGs, the selection and design of the triboelectric layer are critically important. Since this layer largely determines how efficiently charges are generated and stored during the contact electrification process, materials with high charge retention ability are essential [14,15,16]. In particular, materials with high dielectric constant can lead to stronger polarization under external electric field charges, which allows the materials to store more charges rather than improve overall energy conversion [9,17,18,19]. In addition, the dielectric behavior of a triboelectric layer is influenced by factors such as interfacial polarization, interactions between fillers and the polymer matrix, mobile layer position, and the internal structure of the composite [20,21]. Therefore, various strategies, including polymer engineering that is the incorporation of high-k inorganic fillers, and the addition of conductive or semiconductive nanomaterials have been widely explored to ultimately improve the dielectric properties of triboelectric materials and increase the charge density generated in TENGs [22,23,24,25].

To increase the dielectric constant of triboelectric materials in particular, a wide range of fillers has been incorporated into polymer matrices, including high-k inorganic materials such as BaTiO_3_ [26,27,28], SrTiO_3_ [29,30], and TiO_2_ [31,32,33], as well as conductive additives such as metal particles [34,35], metal nanowires [12,36], carbon nanotubes (CNTs) [37,38,39], MXene [40], Metal–organic frameworks (MOFs) [41], and graphene [42,43]. These conductive fillers can act as micro-capacitor structures within the polymer due to the substantial conductivity difference between the polymer matrix and the filler. This effect promotes strong interfacial polarization, facilitates efficient charge accumulation, and ultimately enhances the dielectric constant and surface charge density of the composite, thereby improving TENG output performance [44]. Despite these benefits, the use of conductive fillers presents inherent limitations. When their loading fraction exceeds a critical threshold, continuous conductive pathways are formed in the composite, which accelerates change dissipation and increases leakage current, leading to a noticeable degradation in TENG performance. Additionally, conventional fillers can trigger nucleation in the polymer matrix, leading to brittle mechanical characteristics, while their aggregation further decreases the interfacial area and suppresses interfacial polarization [45,46]. These challenges highlight the need for alternative filler system that can provide high interfacial polarization without generating undesired continuous conductive networks.

To address the suggested limitations, we propose a strategy that uses gallium-based liquid metal particle (LMP) encapsulated by graphene oxide (GO) first to construct a spontaneous redox-driven LMP–GO core–shell structure, following the addition into a hydrogel matrix. Our preliminary study demonstrated that LMP, even at the nanoscale, sparsely triggers a nucleation event when embedded within a polymer matrix [47]. However, LMP readily forms an oxide layer even under low oxygen levels, and the resulting insulating shell significantly diminishes the electrical performance of the LMP core. Here, we demonstrated a redox-driven interfacial strategy in which GO interacts with the LMP surface. The difference in reduction potentials induces the reductive conversion of GO, yielding a reduced GO (rGO) shell that conformally encapsulates the particle surface. Simultaneously, the native oxide layer on the LMP is chemically removed during the redox process, thereby addressing the aforementioned limitation above.

This LMP@rGO structure enhances colloidal stability by increasing electrostatic repulsion among particles. The structure also provides a well-regulated electrical conductivity that originates from the resistive barrier established at the LMP@rGO formed via a spontaneous redox reaction interface together with the intrinsic narrow band gap semiconductive characteristics of Ga-based LMPs [48,49]. This controlled conductivity prevents the rapid development of conductive pathways commonly observed in composites containing conventional conductive fillers. Simultaneously, the core–shell configuration facilitates strong interfacial polarization, allowing the structure to accumulate a high density of triboelectric charges without sacrificing electrical insulation [50]. This balance between moderated conductivity and enhanced polarization overcomes the typical limitations associated with traditional conductive-filler systems. The formation of conductive pathways and the prevention of aggregation-induced performance degradation are critical to the system’s efficiency. Consequently, these LMP@rGO-based hydrogel composites maintain material flexibility while achieving energy conversion efficiencies that single-component filler systems cannot reach. By incorporating LMP@rGO with narrow bandgap semiconductor characteristics, the composites enable a substantial enhancement in the practical output performance of TENG devices.

In this work, we incorporate the redox-driven LMP@rGO core–shell particles into a poly(acrylic acid) (PAA) hydrogel matrix to develop an LMP@rGO/PAA triboelectric layer with tailored electrical resistance, enhanced dielectric properties, and stationary mechanical compliance. Compared with conventional solid-state typical TENGs, hydrogel-based TENG exhibits distinct charge storage and transport mechanisms dominated by ionic dissociation and ion migration within the hydrated polymer network, which can be illustrated in previous studies [51,52]. This ionic nature enables volumetric charge storage and a higher effective dielectric constant, leading to improved charge retention. Moreover, the inherent softness of hydrogels allows conformal contact with opposing triboelectric layers, enabling stable electrical output under low-frequency and low-amplitude mechanical stimuli, such as human motion. The hydrated structure also provides enhanced tolerance to humid environments; thus, the hydrogel-based TENG is particularly attractive for wearable and soft electronic applications.

In this system, incorporating semiconductive LMP@rGO core–shell structures into the PAA hydrogel matrix introduces interfacial and ionic polarization mechanisms that offer a distinct advantage over the conventional hydrogel-based TENGs. The semiconductive fillers suppress metallic conductive pathways while generating localized electric fields at the filler–hydrogel interfaces, which promote ionic dissociation and enhance ionic polarization [53,54]. Consequently, the dielectric constant of the composite hydrogel increases with LMP@rGO content due to strengthened ionic dissociation-induced polarization rather than enhanced electronic conductivity. Under low-frequency excitation, this enhanced polarization improves surface charge retention, while the moderated conductivity of the fillers restricts long-range charge migration, minimizing leakage current and enabling improved triboelectric output.

The fabricated device exhibits sufficient power generation to operate light-emitting diodes (LEDs), demonstrating the practical feasibility of the LMP@rGO/PAA composite. Overall, these results show the potential of LMP@rGO-based hydrogel composites as high performance triboelectric layer materials for next-generation wearable and self-powered electronic system.

## 2. Results and Discussion

**Figure 1:**
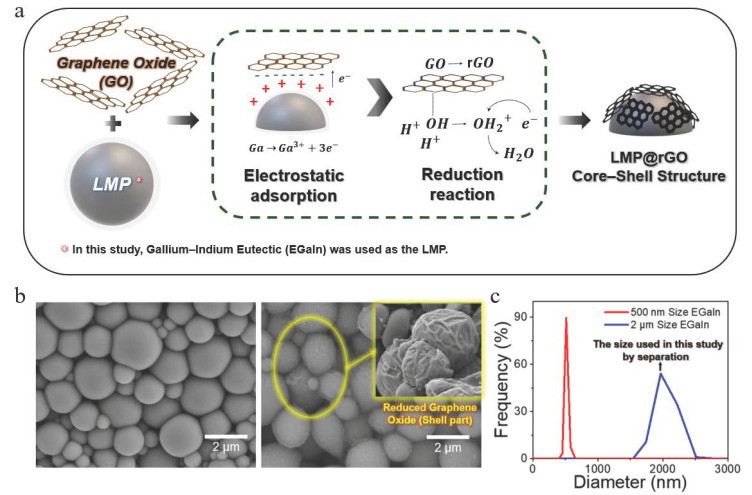
Preparation of LMP@rGO core–shell structure. (**a**) Schematic illustration of mechanism to synthesize LMP@rGO structure form oxidized LMP and GO. (**b**) SEM images of bare LMPs (**left**) and LMP@rGO (**right**). (**c**) Representative particle size distribution of LMP@rGO.

The formation mechanism of the LMP@rGO core–shell structure is dominated by the combined effects of electrostatic adsorption in aqueous media and a subsequent interfacial redox reaction between graphene oxide (GO) and eutectic gallium–indium (EGaIn). In aqueous solution, GO carries a negative surface charge due to its oxygen-containing functional groups, such as hydroxyl (–OH) and carboxyl (–COOH). In contrast, EGaIn develops a positively charged surface through the presence of gallium ions at the metal–liquid interface. This electrostatic charge difference drives the initial electrostatic attraction between GO sheets and EGaIn droplets. Once in contact, gallium atoms, possessing a low reduction potential due to their valence electron configuration, facilitate the reductive removal of oxygen functionalities on GO [55]. This interfacial electron transfer leads to the spontaneous reduction in GO and the formation of Ga–O–C linkages, resulting in a conformal rGO shell around the LMP core. Through this combined adsorption and redox-driven process, the LMP@rGO core–shell structure is spontaneously established, as illustrated in Figure 1a.

Using the interfacial redox reaction, we successfully formed the LMP@rGO core–shell structure, as confirmed by the SEM image in Figure 1b. The visual evidence clearly reveals a wrinkled rGO layer conformally attached to the surface of the spherical LMP (Figure 1b right, compared to bare LMP on left), verifying the formation of a stable core–shell morphology. Here, based on our preliminary studies and structural optimization considerations, and to enable reliable performance in subsequent applications, LMPs were dispersed with an optimized average diameter of approximately 2 μm to ensure robust shell formation. For instance, we confirmed that smaller LMPs, especially those under 500 nm scale, closely match or fall below the lateral dimensions of GO sheets. Under these conditions, our observations exhibited that the LMPs preferentially adsorb onto the GO surface rather than being fully encapsulated, resulting in an inverted or incomplete configuration instead of a stable core–shell structure. LMPs with diameters of near 2 μm were therefore employed to reliably achieve the desired LMP@rGO core–shell structure (Figure 1c).

**Figure 2:**
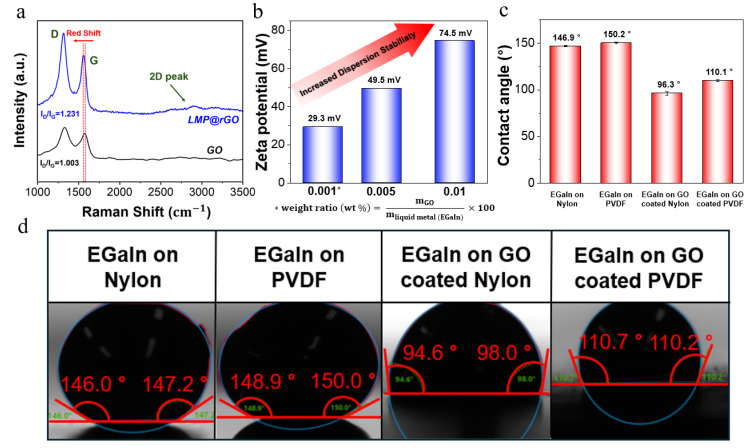
Surface charge characterization of LMPs@rGO. (**a**) Raman spectroscopy of GO and LMP@rGO. (**b**) Zeta potential change with the GO contents increased. (**c**) Contact angle results. (**d**) Each image of LMP(EGaIn) on different substrates.

To verify the interfacial redox reactions and subsequent changes in electronic structure during the synthesis of LMP@rGO through the interaction between GO and EGaIn, we performed Raman spectroscopy analysis. The increase in the $I_{D}/I_{G}$ ratio (from 1.003 to 1.231) signifies the removal of oxygen-containing functional groups, indicating the reduction in GO and the simultaneous formation of numerous micro defects or small ${Sp}^{2}$ domains within the carbon lattice. This serves as direct evidence that EGaIn spontaneously reduced GO to rGO without the need for an external reducing agent. Two-dimensional peak of LMP@rGO also supports the reduction results. Furthermore, a noticeable red shift in the G peak (from 1577 to 1567 ${cm}^{-1}$) was observed, suggesting a change in the charge state of the rGO shell. This shift is likely attributed to the intercalation of gallium ions during the ultrasonication process. These results demonstrate that the LMP core and rGO shell are not merely in physical contact but are strongly coupled through chemical and electronic interactions, leading to a significant modulation of the electronic structure (Figure 2a) [56].

In the case of LMP@rGO particles, where the LMP core is encapsulated by subsequent rGO, stable dispersion requires that the interparticle electrostatic repulsion overcome the inherent Van der Waals attraction. The zeta potential serves as a key indicator of the electrostatic repulsive force within the LMP@rGO system. Under fixed-pH aqueous conditions, an increase in the absolute value of the zeta potential reflects a higher effective surface charge density, indicating stronger electrostatic repulsion between particles. This enhanced repulsion raises the energy barrier for interparticle collisions, thereby effectively suppressing aggregation and improving colloidal stability [57,58].

We also observed that zeta potential increased with higher introducing GO content, indicating that additional GO facilitates the formation of more complete LMP@rGO core–shell structures. Because larger zeta potential magnitudes correspond to improved dispersion stability, this trend confirms that increased core–shell formation directly enhances the stability of the LMP@rGO dispersions (Figure 2b).

To further verify the interfacial interaction between EGaIn (LMP materials) and GO, we performed contact-angle measurements, as shown in Figure 2c,d. When a bare EGaIn was placed on hydrophilic-dominating nylon and hydrophobic PVDF filters, the EGaIn exhibited a very high contact angle of approximately 150 °, which is consistent with the high surface tension and spontaneously formed rigid oxide skin (Ga_2_O_3_), characteristic of oxidized gallium [59,60]. Typically, the formed oxide skin showed very high contact angle, over 150 °C, due to its non-wetting characteristics [61,62]. In contrast, when the same substrates were coated with GO, following the performance of the same test, the contact angles decreased markedly to 96.34° on nylon and 110.07° on PVDF. This substantial reduction demonstrates that GO effectively lowers the interfacial energy of EGaIn through surface redox interactions, thereby enhancing its wettability. These results provide direct experimental evidence of the strong interfacial affinity between EGaIn, ultimately LMP and GO.

Similarly to millimeter-scale LMPs, micro-scale LMPs are expected to exhibit interfacial behaviors consistent with contact angle data, given their spontaneous bonding through redox reactions. Furthermore, the micro-scale system possesses a significantly higher surface-to-volume ratio compared to its millimeter-scale counterpart. This substantially elevated ratio in the micro-scale regime is anticipated to amplify the interactions between the LMP and rGO at the 2D interface, consequently leading to a more positive impact on the overall material performance.

**Figure 3:**
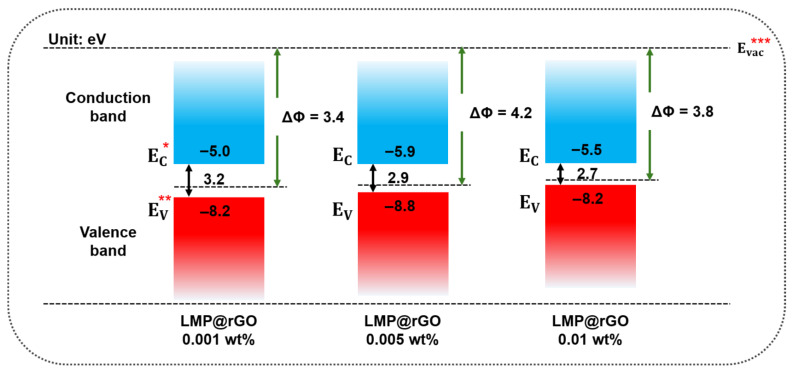
Electrical performance of LMPs@rGO core–shell structure. Electrical bandgap diagram of LMP@rGO structure with the GO contents increase. E_C_ *: conduction band minimum. E_V_ **: valence band maximum. E_Vac_ ***: vacuum energy level.

To elucidate the electrical characteristics of LMP@rGO and evaluate whether the core–shell structure provides the moderated conductivity necessary for suppressing leakage pathways, we performed comprehensive bandgap measurements using ultraviolet photoelectron spectroscopy (UPS) and near-infrared (NIR) spectroscopy. As discussed above, excessively conductive fillers can form conductive networks within polymer composites, leading to increased leakage current and reduced dielectric performance. Thus, characterizing the bandgap of LMP@rGO is crucial for verifying that the redox-inducing core–shell structure achieves the desired semiconductive behavior rather than functioning as a purely insulating or metallic component. Although both GO and oxide skin of LMPs are intrinsically insulating prior to the redox interaction, the resulting LMP@rGO structure exhibited a semiconductive bandgap (Figure 3). Moreover, consistent with the trend observed in the surface charge characteristics, the bandgap gradually decreased with increasing GO content from 0.001 (3.2 eV) to 0.01 wt% (2.7 eV), reflecting the higher probability of forming complete LMP@rGO core–shell structure at elevated GO loading. This trend is consistent with the fact that both the oxide-free LMP core and the rGO shell possess inherently high electrical conductivity.

**Figure 4:**
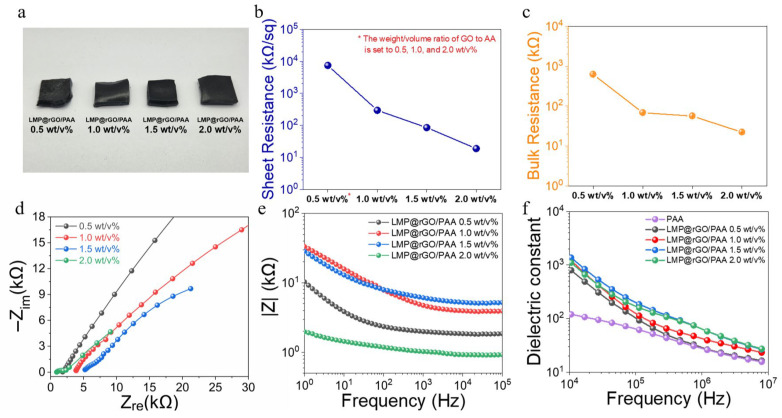
(**a**) Optical image of LMP@rGO/PAA hydrogel and electrical resistance characterization by (**b**) sheet resistance, (**c**) bulk resistance, (**d**) electrochemical resistance, (**e**) Bode plot, and (**f**) dielectric constants. Here, wt/v% in the figures indicates wt/vol%.

To evaluate the applicability of the LMP@rGO structure in TENG devices, we finally prepared triboelectric electrodes by incorporating 0.5 to 2.0 wt/vol% LMP@rGO into a PAA hydrogel matrix (Figure 4a). Prior to verifying the TENG performance, we evaluated both sheet (Figure 4b) and bulk (Figure 4c) resistance with GO loadings to elucidate the electrical transport characteristics of LMP@rGO/PAA composites. A drastic decrease in sheet resistance was observed when the GO-to-AA ratio increased from 0.5 to 1.0 wt/vol%, suggesting that the ionic conduction pathways within the hydrogel matrix began to undergo a transition within this concentration range. At 0.5 wt/vol%, the core–shell structures remain largely isolated within the matrix, resulting in a very high sheet resistance of approximately 7.4 MΩ/sq. However, at 1.0 wt/vol%, a continuous conductive network begins to form, leading to a drop in resistance to ~0.3 MΩ/sq. After the formation of conductive pathways, additional GO incorporation does not significantly affect resistance. Bulk resistance measurements, reflecting three-dimensional electrical performance, also displayed an identical trend, further verifying that the ionic conduction pathways within the hydrogel underwent a transition within the 0.5–1.0 wt/vol% range. The consistency between the sheet and bulk resistance trend indicates that the LMP@rGO structures form an isotropic three-dimensional conductive network throughout the hydrogel, rather than being confined to a two-dimensional plane. This observation also corroborates the homogeneous distribution of the core–shell structures within the PAA matrix.

For triboelectric application, lastly, high surface charge capacity is necessary. Nyquist plots of the LMP@rGO/PAA hydrogels can effectively reveal a progressive decrease in both charge transfer resistance and ionic diffusion impedance with introduced GO content increasing (Figure 4d). The Nyquist plots revealed that the hydrogel exhibits a solution resistance of approximately 2.0 kΩ at a GO-to-AA ratio of 2.0 wt/vol%. As the GO content decreases, the solution resistance increases steadily, and at a loading of 0.5 wt/vol% or below, it becomes difficult to reliably determine the resistance, indicating the absence of proper ionic transport pathways and the emergence of a semi-insulating state.

When the loading of semiconductive particles exceeds a certain level, the hydrogel typically undergoes a structural transition in its internal water organization, which promotes the formation of continuous and dynamically connectable ionic transport pathways. This transition significantly enhances ionic conductivity by enabling long-range ion migration through interconnected hydration channels [54,63]. As a result, an abrupt decrease in the real component of impedance is observed in the Nyquist plots. In addition, this behavior originates from ionic percolation associated with water channel connectivity rather than from electronic percolation through conductive filler networks, as widely reported for ionically conductive hydrogel systems [64].

The electrochemical impedance hence results in exhibiting a transition of ion conductivity that is fully consistent with the electrical resistance measurements, collectively confirming that the transition in charge transport behavior occurs within the GO loading range of 0.5–1.0 wt/vol%.

In the frequency range above $10^{4}\;$Hz from Bode plot, the impedance magnitude shows a frequency-independent plateau. This indicates that beyond this frequency, charges no longer accumulate at the interfaces. Instead, the system acts as a resistor where ions and charges flow freely through the electrochemically conductive network of the hydrogel (Figure 4e). To further validate these observations, we measured the dielectric permittivity over a frequency range of kHz to MHz. In the low-frequency region, ions and charges effectively respond to the external electric field and are stored in the form of polarization.

However, in the high-frequency region, they exhibit Maxwell–Wagner–Sillars (MWS) relaxation behavior, where they fail to synchronize with the rapid oscillations of the electric field, leading to conduction rather than polarization. [65] The dielectric constant result reveals that the permittivity remains relatively higher as the GO content increases, which is attributed to the increased number of rGO sheets acting as micro-capacitors within the PAA matrix. Since capacitance is inversely proportional to the distance between electrodes (rGO sheets in this study), the increased rGO content reduces the inter-sheet distance, thereby enhancing the capacitance. Consequently, while the average dielectric constant of pure PAA was measured at approximately 19.8 in the high-frequency range of 1.2 MHz to 8.0 MHz, the 2.0 wt/vol% sample achieved the highest dielectric constant of 40.2 (Figure 4f).

**Figure 5:**
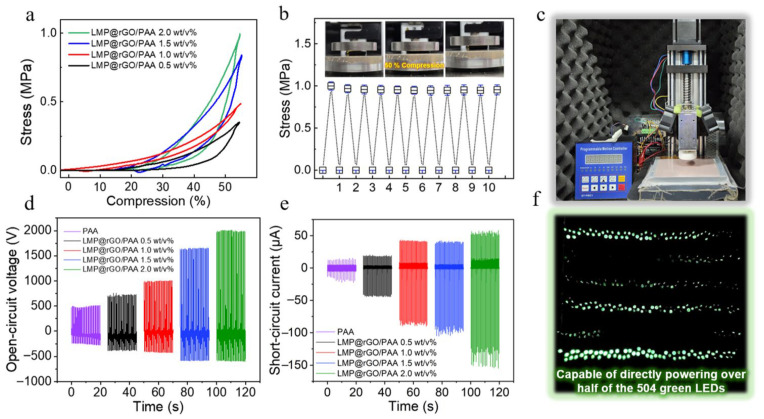
Compressive properties of LMP@rGO/PAA hydrogels and the output performance of the resulting TENG. (**a**) Compression stress of the LMP@rGO/PAA hydrogels with compressions. (**b**) Compression stability of the LMP@rGO/PAA 2.0 wt/vol% for a 10-time cycle with 50% compression. (**c**) Optical image of TENG device. (**d**) Open-circuit voltage. (**e**) Short-circuit current. (**f**) Image of green LEDs illuminated by the TENG. Here, wt/v% in the figures indicates wt/vol%.

To demonstrate that challenges such as hydrogel dehydration and ion migration can be mitigated through the use of semiconductive additives, we conducted compression tests. The incorporation of semiconductive additives, particularly the rGO surfaces, contributes to enhanced water retention within the PAA hydrogel, thereby delaying dehydration, as clearly evidenced by the mechanical characterization (Figure 5a). Among the samples, the LMP@rGO/PAA 2.0 wt/vol%, which contains the highest filler loading, maintained its mechanical strength even after ten repeated compression cycles at over 50% strain (Figure 5b). Furthermore, it preserved its structural volume (xyz dimensions) when exposed to laboratory conditions (23 °C, 45% RH) for one week. We also confirmed that the compressive modulus of the LMP@rGO/PAA hydrogel with 2.0 wt/vol% loading is 1018 ± 14.7 kPa, indicating that the developed hydrogels exhibit a soft yet mechanically robust nature, assisted by the presence of the liquid metal body.

To evaluate the TENG performance of the LMP@rGO/PAA hydrogels, we utilized a custom-built TENG device (Figure 5c). The TENG performance results of LMP@rGO/PAA hydrogels exhibited a clear enhancement in open-circuit voltage (Figure 5d) and short-circuit current (Figure 5e) with increasing GO content, demonstrating progressively improved triboelectric performance. This improvement can be attributed to chemical interactions between the rGO shell and the PAA matrix. Specifically, hydrogen bonding between the oxygen-containing functional groups of GO (–OH, –COOH) and PAA enhances the mechanical robustness of the hydrogel and increases the amount of bound water and stabilizes the structural framework. Also, the incorporation of LMPs helps the hydrogel maintain mechanically stable softness, which enhances conformal contact during friction motion and further contributes to improved charge generation that is demonstrated by our previous studies [47,66].

Consistent with the electrical output results, the dielectric constant of the LMP@rGO/PAA hydrogel also increased with higher GO loading, confirming the direct correlation between interfacial interactions, dielectric enhancement, and the observed TENG performance. A higher dielectric constant effectively reduces the coulombic attraction between ions within the hydrogel. This weakened coulomb force prevents ionic aggregation and consequently allows the charge-carrying ions to remain in a more dissociated and stabilized state. Such ionic stabilization enhances the ability of the triboelectric layer to retain surface charges, thereby improving energy-harvesting efficiency. Finally, an increase in dielectric constant enables a greater amount of charge to be stored on the surface, which is consistent with the observed trends in open-circuit voltage and short-circuit current. When the LMP@rGO/PAA hydrogel containing 2.0 wt/vol% filler was tested in a TENG device, more than half of the 504 green LEDs connected in series were illuminated, demonstrating the practical energy-harvesting capability of the material (Figure 5f, Video S1).

## 3. Conclusions

We successfully prepared LMP@rGO core–shell structures through a spontaneous redox reaction between oxide skin on LMPs and GO, effectively overcoming the intrinsic electrical limitations of both materials. Increasing the GO content enhanced the dispersion stability and moderated electrical conductivity of the composite, as confirmed by the increased zeta potential and the existence of a semiconductive bandgap of approximately 2.7 eV. After incorporating into a PAA hydrogel, the LMP@rGO structures formed extensive hydrogel-bonding networks with swelled PAA, offering high surface charge retention while maintaining mechanical softness suitable for triboelectric applications. The resulting LMP@rGO/PAA hydrogels showed improved TENG performance with higher GO content, as following increases in open-circuit voltage, short-circuit current, and dielectric constant. Moreover, the TENG including the 2.0 wt/vol% LMP@rGO/PAA hydrogel generated sufficient output to illuminate more than half of 504 series-connected green LEDs, representing the practical applicability of the composite. These results highlight the potential of LMP@rGO hydrogel composites as high-performance, flexible triboelectric layer materials for next-generation wearable and self-powered electronic systems.

## 4. Materials and Methods

### 4.1. Preparation of LMP@rGO Core–Shell Structure

The LMP@rGO core–shell structures were synthesized as follows. GO (GO-V50, Standard Graphene, Ulsan, Republic of Korea) dispersion was first prepared by adding 0.5, 2.5, and 5.0 mg of GO to 10 mL of deionized water and 1.1 mL of 1N HCl, using sonication (GTS, Pohang, Republic of Korea) for 15 min. Bulk LM (EGaIn, Goodfellow, San Angelo, TX, USA, Ga: In mass ratio = 75.5:24.5) was dispersed by sonicating 750 mg of LM in 15 mL of deionized water for 10 min. Equal volumes (5 mL each) of the two sonicated suspensions were then combined to obtain a 10 mL mixture, which was rapidly subjected to bath sonication for 10 min to promote the redox-driven formation of LMP@rGO core–shell structures. The resulting dispersion was allowed to cool to room temperature.

### 4.2. Synthesis of LMP@rGO/PAA Hydrogel

LMP@rGO core–shell structures for hydrogel preparation were synthesized using the same procedure described above, except that only the mass ratio between LMP and GO was controlled. For this series, 150 mg of LMP was used, and four GO loadings, 50, 100, 150, and 200 mg, were prepared. To prepare the hydrogels, 10 mL of AA monomer was added to each LMP@rGO dispersion. The original GO-to-AA ratios were set to 0.5, 1.0, 1.5, and 2.0 wt/vol% to obtain samples with different filler contents. The mixtures were mechanically stirred at 250 rpm for 2 min and then immediately transferred to Petri dishes. Gelation was performed by solution casting at 30 °C for approximately 6 h, yielding the LMP@rGO/PAA hydrogels.

### 4.3. Characterization of Morphologies

Field-emission SEM (FE-SEM, JSM-7900F, JEOL, Tokyo, Japan) was used to display the images of bare LMPs and LMP@rGO core–shell structures with 10 kV of accelerate voltage.

### 4.4. Surface Charge Characterization of LMPs@rGO

The surface reduction in LMP@rGO was investigated using a confocal laser Raman spectrometer (NRS-4500, JASCO, Tokyo, Japan). The Raman spectra were acquired using an excitation laser wavelength of 531.92 nm with a laser power of 23.0 mW. Each spectrum was collected with an exposure time of 3 s and 10 accumulations to ensure a high signal-to-noise ratio. The particles size and zeta potential results were obtained using a particle size analyzer (SZ-100V2, HORIBA, Kyoto, Japan). The contact angle of LMPs was measured using a contact angle goniometer (DSA 25S, KRUSS, Hamburg, Germany). For each sample, ten independent measurements were performed, and the mean values with standard deviations were reported.

### 4.5. Electrical Performance of LMPs@rGO Core–Shell Structure

The optical bandgap of the LMP@rGO structure was determined using a NIR spectrophotometer (Cary 5000, Agilent, Santa Clara, CA, USA). The spectra were collected over the range of 200–2500 nm with a step interval of 1 nm and an integration time of 0.1 s per point. The instrument automatically switched the light source at 350 nm and the detector at 800 nm to ensure optimal signal acquisition across the full spectra window. The work function of LMP@rGO was finally determined using ultraviolet photoelectron spectroscopy (UPS, Theta Probe, Thermo Fischer Scientific, Waltham, MA, USA) with a He I UV source (21.2 eV). A −10 eV bias was applied to the sample holder during measurement. Surface cleaning was performed by Ar^+^ sputtering at 1 keV for 30 s, corresponding to an estimated removal depth of −0.05 nm (TaO calibration). The sputtering was carried out over a raster area of 10 mm × 10 mm.

### 4.6. Characterization of Electrical Resistance LMP@rGO/PAA Hydrogel

The electrical properties of the LMP@rGO/PAA hydrogel were characterized using samples with a fixed area of 4 cm × 4 cm and a thickness of 3.5 mm. The sheet resistance was measured using two four-point probes (CMT-100S, Advanced Instrument Technology, South Korea, and four-point-probe original, Ossila, Sheffield, UK) to ensure data reliability. The bulk resistance was measured using a multimeter (34465A, Keysight, Santa Rosa, CA, USA). Electrochemical impedance spectroscopy (EIS), including Nyquist and Bode plots, was performed using a Potentiostat (VersaSTAT 3, Princeton Applied Research, Oak Ridge, TN, USA) via VersaStudio software with a 10 mV RMS amplitude over a frequency range of 0.1 Hz to 100 kHz. This measurement employed a three-electrode system consisting of an Ag/AgCl (in 3 M KCl) reference electrode, a platinum mesh counter electrode, and the hydrogel as a working electrode with an effective area of 1 cm × 1 cm. Additionally, the dielectric constant was measured using an LCR meter (IM 3536, HIOKI, Nagano, Japan) in the range of 1 kHz to 8 MHz at 21 °C and 23% humidity, maintaining the same sample dimensions of 4 cm × 4 cm in area and 3.5 mm in thickness.

### 4.7. Mechanical Properties of LMP@rGO/PAA Hydrogel

To evaluate the mechanical stability of the LMP@rGO/PAA hydrogel, which is directly related to the output performance of the TENG, compression tests were conducted using a Universal Testing Machine (UTM, MCT-1150, A&D, Tokyo, Japan). The LMP@rGO/PAA hydrogel samples, with dimensions of 2 cm × 2 cm and a thickness of 3.5 mm, were tested using a 500 N load cell. The hydrogels were compressed to 52% strain at a constant speed of 10 mm/min, followed by decompression at the same speed. This cyclic compression–decompression process was repeated for 10 cycles to assess the structural durability of the hydrogel.

### 4.8. LMP@rGO/PAA Hydrogel-TENG’s Property Test

TENG performance was evaluated using a custom-built contact separation testing system (specific information was reported in [21]). Polytetrafluoroethylene (PTFE) film was used as the negative triboelectric layer due to its distinct electron-accepting characteristics. The LMP@rGO/PAA hydrogels were assembled into a TENG device using acrylic plates, copper tape, silver paste, and copper wiring, with each device fabricated in a 4 cm × 4 cm active area. Measurements were performed at a frequency of 1 Hz with a separation distance of 3.75 cm. The contact force was measured at 11.0 N at 32 V.

## Data Availability

The data presented in this study are available upon request from the authors.

## Supplementary Materials

The following supporting information can be downloaded at https://www.mdpi.com/article/10.3390/gels12010086/s1, Video S1: Real-time video of powering more than half of the 504 green LEDs.
